# Detection of an Undescribed *Babesia* sp. in Capybaras and *Amblyomma* Ticks in Central-Western Brazil

**DOI:** 10.3390/ani13010094

**Published:** 2022-12-27

**Authors:** Lucianne Cardoso Neves, Lucas Christian de Sousa-Paula, Sarah Alves Dias, Bianca Barbara Fonseca da Silva, Warley Vieira de Freitas Paula, Luiza Gabriella Ferreira de Paula, Brenda Gomes Pereira, Gracielle Teles Pádua, Ana Carolina Borsanelli, Ennya Rafaella Neves Cardoso, Felipe da Silva Krawczak, Filipe Dantas-Torres

**Affiliations:** 1 Veterinary and Animal Science School, Federal University of Goiás, Goiânia 74605-220, Brazil; 2 Laboratory of Immunoparasitology, Department of Immunology, Aggeu Magalhães Institute, Oswaldo Cruz Foundation (Fiocruz), Recife 50740-465, Brazil; 3 Tick-Pathogen Transmission Unit, Laboratory of Bacteriology, National Institute of Allergy and Infectious Diseases, Hamilton, MT 59840, USA

**Keywords:** *Babesia* sp., *Amblyomma sculptum*, *Amblyomma dubitatum*, rodents

## Abstract

**Simple Summary:**

Capybaras are known hosts for various tick species, but there are limited data regarding the tick-borne pathogens they can carry. We assessed the presence of piroplasmids and *Ehrlichia* spp. in capybaras and their associated ticks in Goiás state, central-western Brazil. Neither capybaras nor ticks were positive for *Ehrlichia* spp. However, we detected an undescribed species of protozoan in both the capybaras and ticks. Further research is required for a formal delineation of this protozoan species, as well as to investigate the role of these ticks as vectors and the possible pathogenicity of this parasite to other animals, including horses.

**Abstract:**

Capybaras (*Hydrochoerus hydrochaeris*) are the largest rodents on Earth. While capybaras are hosts for various tick species, there is limited information regarding the tick-borne pathogens they can carry. We investigated the presence of piroplasmids and *Ehrlichia* spp. in capybaras and their associated ticks in two peri-urban areas in Goiás state, central-western Brazil. Blood samples collected from 23 capybaras were used to investigate the presence of piroplasmids and *Ehrlichia* spp. in stained-blood smears and by PCR. Ticks collected from the capybaras were identified morphologically and also tested using PCR for the same pathogens. A total of 955 ticks were collected, including 822 (86.1%) *Amblyomma sculptum*, 132 (13.8%) *Amblyomma dubitatum*, and one (0.1%) unidentified larva of *Amblyomma* sp. Neither the capybaras nor ticks were positive for *Ehrlichia* spp. However, a stained-blood smear examination revealed the presence of ring-stage and pyriform-shaped merozoites in the erythrocytes of one (4.4%) capybara. In the same way, 47.8% (11/23) and 19.9% (36/181) of blood samples and ticks, respectively, were positive for piroplasmids in the PCR. We successfully sequenced a partial 18S rRNA gene fragment of four samples (two capybaras, one *A. sculptum*, and one *A. dubitatum*), and the phylogenetic reconstruction disclosed that the organism reported in the present study clusters within the genus *Babesia*. Further research is required for a formal delineation of this species (designated as *Babesia* sp. strain Capybara) and to investigate the hypothesis of *A. dubitatum* and *A. sculptum* ticks being vectors.

## 1. Introduction

Capybaras (*Hydrochoerus hydrochaeris* (Linnaeus, 1766)) are the biggest rodents on Earth and are primarily associated with forests, seasonally flooded savannas, and wetlands. However, the habitat loss due to the extensive urbanization process in the major Brazilian regions has forced capybaras and many other animals to adapt to living in proximity to humans [1,2], posing risks to themselves, domestic animals, and humans. For instance, they can cause crop damage [3]. If they feel threatened, capybaras can attack other animals and humans, and the consequences of bites can be severe [4,5]. Moreover, they can also carry ticks and tick-borne pathogens that may infect animals and humans [6].

In this regard, capybaras are primary hosts for *Amblyomma sculptum* (Berlese, 1888), the principal vector of *Rickettsia rickettsii* (Wolbach, 1919), the etiological agent of Rocky Mountain spotted fever or Brazilian spotted fever [7,8,9]. Beyond acting as hosts for *A. sculptum*, capybaras serve as a source of *R. rickettsii* infection for ticks, playing an epidemiological role in the transmission of this pathogen in Brazil [7,8,9]. Excluding *Rickettsia* spp., there is scant information about other tick-borne pathogens (e.g., *Anaplasma* spp., *Babesia* spp., *Ehrlichia* spp., and *Theileria* spp.) that could possibly infect capybaras. For instance, Criado-Fornelio et al. [10] detected a piroplasmid (order Piroplasmida) in a capybara from southern Brazil that shared a 90% identity with *Theileria equi* (Laveran, 1901). More recently, Gonçalves et al. [11] detected a piroplasmid in a nymph of *Amblyomma dubitatum* Neumann, 1899, collected from a black rat (*Rattus rattus* Linnaeus, 1758), and in a female of the same tick species collected from a capybara. The obtained 18S rRNA sequences shared, respectively, 99.4% and 97.2% identity, respectively, with the sequence previously reported by Criado-Fornelio et al. [10].

In the present study, we investigated the presence of piroplasmids and *Ehrlichia* spp. in capybaras and their associated ticks in two peri-urban areas in central-western Brazil. We also molecularly characterized a piroplasmid detected in capybaras and their ticks by conducting a comprehensive phylogenetic assessment based on piroplasmid 18S rRNA gene sequences available in GenBank.

## 2. Materials and Methods

### 2.1. Study Area

From July 2020 to April 2022, ticks and blood samples were collected from capybaras (*n* = 23) captured on the campus of the Federal University of Goiás (Site 1) (*n* = 17) (16°35′42″ S, 49°16′50″ W, 718 m altitude) and in a residential park (Site 2) (*n* = 6) (16°36′26″ S, 49°10′27″ W, 844 m altitude), Goiânia City, Goiás, central-western Brazil; five capybaras were recaptured (three recaptured once and two twice).

Other animals—i.e., cattle, coatis [*Nasua nasua* (Linnaeus, 1766)], black-striped capuchin [*Sapajus libidinosus* (Spix, 1823)]—were present in these areas, but we did not collect ticks from them.

### 2.2. Ticks and Blood Sample Collection

Capybaras were captured by using 90 m^2^ corrals baited with corn, corn silage, banana leaves, and sugar cane. The frequency of captures varied according to the year. In particular, captures were carried out weekly from July 2020 to December 2021 and monthly from January to April 2022. Capybaras were physically restrained with a net and anesthetized with an intramuscular injection of ketamine (10 mg/kg) plus xylazine (0.5 mg/kg). Under anesthesia, a 3 mL blood sample was withdrawn from the saphenous vein of each capybara, and a thorough physical was conducted to detect any attached ticks. Capybaras were identified with a subcutaneous microchip (Allflex), clinically monitored during the procedure until recovery from anesthesia, and released at the same capture site. Collected ticks were placed in plastic tubes and sent to the laboratory for species identification, according to the morphological keys for nymphs and adults [12,13]. Because there is no taxonomic key for the larvae of Brazilian *Amblyomma* spp., larvae were identified on the genus level only [14].

### 2.3. Blood Smear, DNA Extraction, and PCR Testing

Blood smears were prepared immediately after blood collection and stained with a rapid stain kit (Panótico Rápido, Laborclin, Brazil) for cytological examination under a standard light microscope.

DNA from whole blood samples (200 µL) was extracted using DNeasy^®^ Blood and Tissue Kit (Qiagen, Valencia, CA, USA) following the manufacturer’s instructions. Tick DNA was extracted individually using the guanidine isothiocyanate protocol for adults [15] and the boiling protocol for nymphs [16].

Extracted DNA from ticks and blood samples were tested using conventional PCR protocols targeting a 378 bp fragment of the *Ehrlichia* spp. The *dsb* gene (forward primer: TTGCAAAATGATGTCTGAAGATATGAAACA; reverse primer: GCTGCTCCACCAATAAATGTATCYCCTA) [17] and a 551 bp fragment of the 18S rRNA gene of piroplasmids (forward primer: CCGTGCTAATTGTAGGGCTAATACA; reverse primer: GCTTGAAACACTCTARTTTTCTCAAAG) [18]. Negative DNA samples were further tested using PCR protocols targeting the 16S rDNA gene of ticks or the cytochrome *b* gene of mammals [19,20] in order to validate the DNA extraction protocol.

### 2.4. DNA Sequencing and Bioinformatic Analyses

PCR amplicons were purified using the SV Gel and PCR Clean-Up System kit (Wizard, Madison, WI, USA) according to the manufacturer’s instructions. Then, amplicons were prepared using the BigDye terminator v3.1 matrix standard kit (Applied Biosystems, Foster City, CA, USA), and forward and reverse sequencing reactions were carried out in a 3500× L genetic analyzer (Applied Biosystems, Foster City, CA, USA) using the same primers as for the PCR. The yielded reads were analyzed using the Staden package [21], and contig assembly was performed using sense and antisense reads based on a Phred quality score ≥ 30. Sequence similarity searching was performed using the Basic Local Alignment Search Tool (BLASTn) (http://blast.ncbi.nlm.nih.gov/Blast.cgi (accessed on 2 November 2022)) and Piroplasmida (taxid:5863) database.

Sequence alignment was performed using the MAFFT algorithm [22], approaching the integrative refinement method FFT-NS-i. Phylogenetic analysis was performed as described elsewhere [23]. In brief, phylogenetic reconstruction was performed using maximum likelihood inference using IQ-TREE version 1.6.12 [24] approaching an ultrafast bootstrap (1000 replicates) method. The best-fit evolutive model was calculated using ModelFinder [25], implemented in IQ-TREE, and selected according to the Bayesian Information Criterion (BIC). The ML tree was visualized and edited using the iTOL v.4 tool [26]. Genetic distances were calculated using MEGA7 [27] between intra- and interspecific groups (i.e., between the *Babesia* sensu stricto clade and the clade encompassing parasites detected in rodents from Brazil and the organism reported in the present study).

## 3. Results

A total of 955 ticks were collected, including 822 (86.1%) *A. sculptum* (460 nymphs, 236 males, and 126 females), 132 (13.8%) *A. dubitatum* (45 nymphs, 42 males, and 45 females), and one (0.1%) unidentified larva of *Amblyomma* sp. (Table 1). *Amblyomma sculptum* predominated in both sites, representing 83.7% and 97.6% of the ticks collected on the university campus (Site 1) and in the residential park (Site 2), respectively.

Overall, 181 ticks were used for DNA extraction, including 149 adults (66 females and 83 males) and six nymphs of *A. sculptum* and 26 adults (11 females and 15 males) of *A. dubitatum*. None of the ticks contained *Ehrlichia* spp. DNA. On the other hand, a Piroplasmida 18S rRNA gene fragment was successfully amplified from 19.9% (36/181) of the ticks, 66.7% (24/36) of which were *A. sculptum* (13 females and 11 males), and 33.3% (12/36) were *A. dubitatum* (three females and nine males).

**Figure 1:**
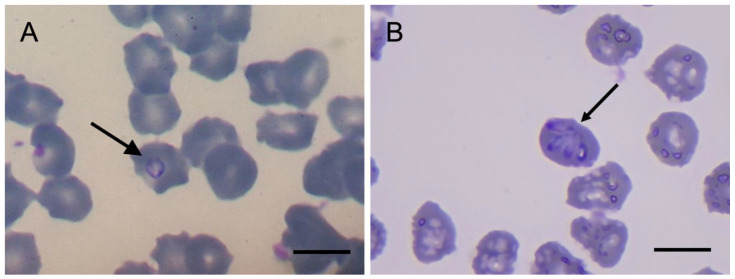
Intra-erythrocytic inclusions (arrowed) found in one of the infected capybaras. (**A**), a ring stage. (**B**), pyriform shape merozoites (note: the whitish structure over one of the parasites is a staining artifact). Scalebars = 10 µm.

Stained-blood smear examinations revealed intra-erythrocytic inclusions similar to the trophozoite (ring stage) and merozoites (pyriform shape) of *Babesia* sp. in one (4.4%) capybara (Figure 1). All capybaras were negative for *Ehrlichia* spp. However, 11 samples (39.3; *n* = 28; two samples from animals recaptured twice were not tested by PCR) were positive for the Piroplasmida 18S rRNA gene fragment, all of which were captured at the university campus (Site 1). All DNA samples from ticks and blood samples that had negative results for piroplasmids and *Ehrlichia* spp. were positive for the 16S rDNA gene of ticks and the cytochrome *b* gene of vertebrates, respectively.

We successfully sequenced the Piroplasmida 18S rRNA gene fragment from two capybaras and two female ticks (one *A. sculptum* and one *A. dubitatum*); other positive samples contained a very low amount of DNA, and sequencing was not possible. The four sequences produced were identical to each other, and a consensus sequence was deposited in GenBank under the accession number OP714347. The BLAST search revealed that the organism detected herein presented a high identity (99.3–100%) with two sequences referred to as *Babesia* sp., available in GenBank (accession numbers: EF222255 and MW290045).

**Figure 2:**
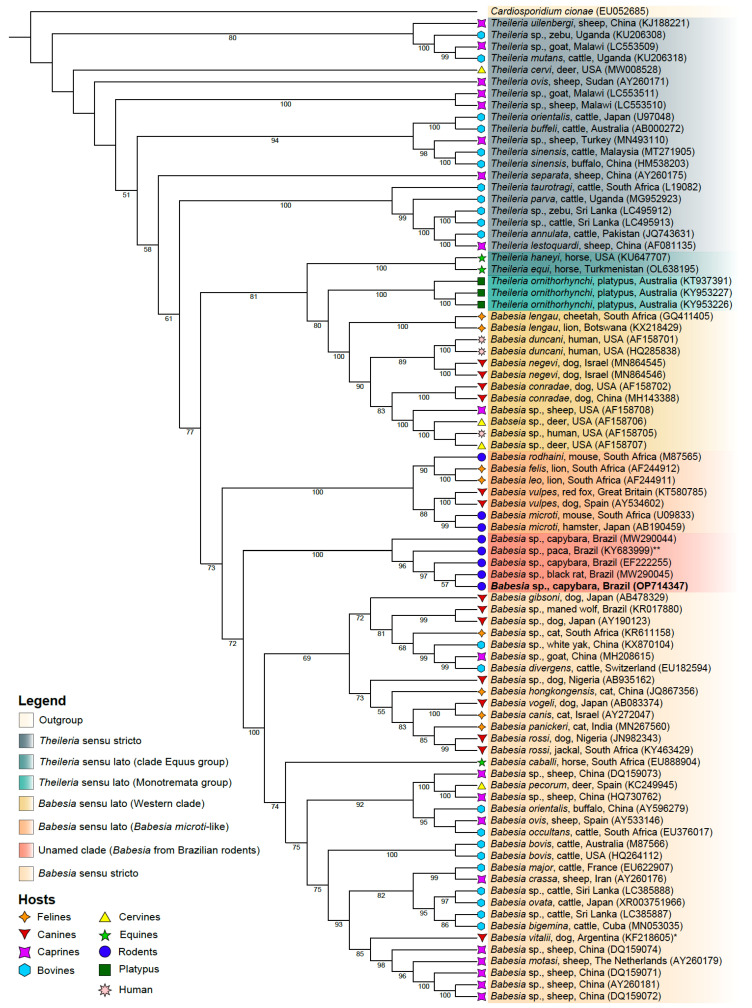
Phylogenetic reconstruction of *Theileria* and *Babesia* species based on a 610-bp alignment of the 18S rRNA gene. Maximum likelihood tree inferred using TN + F + I + G4 evolutive model. The support of branches above 50 is shown (based on 1000 bootstrap replicates). Symbols at branch tips refer to the hosts from which parasites were detected. The sequence generated in the present study is highlighted in bold. The 18S rRNA gene sequence (EU052685) of *Cardiosporidium cionae* (Van Gaver and Stephan, 1907) was used as an outgroup. Sequences marked * or ** are referred to as *Rangelia vitalii* (Pestana, 1910) and *Theileria* sp. in GenBank, respectively. The nomenclature of the clades is according to Jalovecka et al. [28].

Our phylogenetic reconstruction, using sequences available for numerous *Babesia* and *Theileria* spp., disclosed that the organism reported in the present study can be placed within a clade (branch support = 100%) that includes other parasites detected in rodents from Brazil (i.e., capybara, paca, and rat) (Figure 2). This clade shares a common ancestor (branch support = 72%) with the *Babesia* sensu stricto clade. As such, the organism detected in capybaras reported in the present study was designated *Babesia* sp. strain Capybara. In addition, our analyses revealed a high inter-clade distance (11.5%) between these two clades in contrast to the intra-clade distance observed within the *Babesia* sensu stricto clade (5.4%) and within the clade comprising parasites detected in rodents in Brazil (~1.0%).

## 4. Discussion

We reported the presence of a piroplasmid parasite infecting capybaras, *A. sculptum*, and *A. dubitatum* in central-western Brazil. Interestingly, 64.7% (11/17) of the capybaras captured at the university campus were positive for this organism, whereas all capybaras from the residential park were negative. Possible explanations for this difference could be the low number of capybaras captured and the comparatively low level of tick infestation in the residential park. Another aspect to consider was the higher number of *A. dubitatum* collected at the university campus as compared with the residential park. Nonetheless, this does not seem to be a factor in explaining the absence of the piroplasmid in capybaras from the residential park, as ticks from both species were positive for the parasite detected in the capybaras.

Morphologically, the parasite detected herein resembles a large *Babesia*. BLAST similarity and phylogenetic analyses support its inclusion in the genus *Babesia*, particularly in a clade containing other undescribed parasites found infecting rodents in other Brazilian states [10]. The phylogenetic relationship of piroplasmids is still an open question, with both *Babesia* and *Theileria* genera segregating into several clades each [28]. In fact, the current knowledge suggests that host interaction may be the major driving force of piroplasmid diversification [28]. The fragment of the 18S RNA gene amplified herein offers a glimpse into the phylogeny of this clade of undescribed parasites from rodents in Brazil, which appears to be related to *Babesia* sensu stricto [29,30]. However, further research is needed to determine the phylogenetic position of this species, which was also discussed by Criado-Fornelio et al. [10].

As such, it is plausible to suppose these organisms represent a putative novel *Babesia* sp. that parasites capybaras and other rodents in Brazil. An organism designated “*Babesia* sp. isolate LR5” (GenBank accession number: MW290045) (100% similarity with *Babesia* sp. strain Capybara) was detected from an infected *A. dubitatum* tick found on a black rat. Our data reinforce the putative role of *A. dubitatum* as a vector of *Babesia* sp. strain Capybara. *Amblyomma dubitatum*, popularly known as the capybara tick, is well adapted to wet environments and typically feeds on capybaras, although different developmental stages of this tick may infest other animals, including small rodents and birds [31,32,33].

*Amblyomma sculptum* is another tick commonly found on capybaras in Brazil, being the primary vector of *R. rickettsii* in Brazil [6]. Altogether, our data suggest that both *A. dubitatum* and *A. sculptum* could play a role as vectors of *Babesia* sp. strain Capybara in the regions where this parasite may be found. This hypothesis deserves further research, especially considering that some positive ticks found in this study were feeding on negative capybaras. Finally, further research is needed to investigate whether *Babesia* sp. strain Capybara is pathogenic to capybaras or to other animals, such as horses, which are frequently parasitized by these ticks.

## 5. Conclusions

The present study reports on the presence of an undescribed parasite (*Babesia* sp. strain Capybara) in capybaras in central-western Brazil. Further research is required for a formal delineation of this species and to investigate the hypothesis of *A. dubitatum* and *A. sculptum* ticks as vectors of this parasite.

## Figures and Tables

**Table 1:** Number of tick-infested and piroplasmid-positive capybaras according to each collection site. The number and species of ticks identified, as well as the number of positive ticks, is also shown.

Site	Number of Capybaras Examined (*n*)	Number of Piroplasmid-Positive Capybaras (*n*, %)	Number of Tick-Infested Capybaras (*n*, %)	Number of Ticks Collected (*n*)			Number of Ticks Tested (*n*)/Number of Piroplasmid-Positive Ticks (*n*, %)		Total Number of Ticks Tested/Total Number Piroplasmid-Positive Ticks (*n*, %)
				*Amblyomma sculptum*	*Amblyomma dubitatum*	*Amblyomma* sp.	*Amblyomma sculptum*	*Amblyomma dubitatum*	
Site 1	17	11, 64.7%	17, 100%	656	128	1	99/19, 19.2%	26/12, 46.1%	125/31, 24.8%
Site 2	6	0, 0%	6, 100%	166	4	0	56/5, 8.9%	0/0, 0%	56/5, 8.9%
Total	23	11, 47.8%	23, 100%	822	132	1	155/24, 15.4%	26/12, 46.1%	181/36, 19.9%

## Data Availability

The data generated or analyzed during this study are included in this published case study. The consensus sequence generated in this study is available in GenBank (OP714347).
